# Proximal vs. total gastrectomy for proximal advanced gastric cancer: a systematic review and meta-analysis of propensity score-matched studies

**DOI:** 10.3389/fonc.2025.1632011

**Published:** 2025-09-26

**Authors:** Chengcong Liu, Yanlei Li, Yan Liang, Zhen Tian, Lin Zhang

**Affiliations:** ^1^ Department of General Surgery, Qingdao Central Hospital, University of Health and Rehabilitation Sciences, Qingdao, China; ^2^ Department of Oncology, Qingdao Central Hospital, University of Health and Rehabilitation Sciences, Qingdao, China; ^3^ Department of General Practice, Qingdao Central Hospital, University of Health and Rehabilitation Sciences, Qingdao, China; ^4^ Medical School, Nanjing University, Nanjing, China

**Keywords:** proximal gastric cancer, locally advanced gastric cancer, proximal gastrectomy, total gastrectomy, propensity score matching, meta-analysis

## Abstract

**Background:**

The optimal surgical approach for locally advanced proximal gastric cancer (LAPGC) remains controversial. While total gastrectomy (TG) is widely accepted, proximal gastrectomy (PG) is increasingly considered to preserve function. This study represents the first meta-analysis to comprehensively compare the surgical and oncological outcomes of PG versus TG for LAPGC using data from propensity score-matched (PSM) studies, addressing a critical gap in surgical decision-making.

**Methods:**

A comprehensive search of various electronic databases was conducted. Studies comparing PG and TG in LAPGC with PSM methodology were included. Pooled hazard ratios (HRs), odds ratio (OR) and mean difference (MD) with 95% confidence intervals (CI) were calculated using a random-effects model. Primary outcomes were overall survival (OS) and disease-free survival (DFS). Secondary outcomes included surgical metrics and postoperative complications.

**Results:**

A total of 265 articles were screened, and five retrospective studies were included in this meta-analysis, comprising 412 patients after PSM. Surgical approaches (OR 1.03, P = 0.896), positive surgical margins (OR 2.83, P = 0.08), and adjuvant chemotherapy rates (OR 1.07, P = 0.19) were similar between the PG and TG groups. PG resulted in significantly shorter operative times (MD 25.7, P<0.001) but higher blood loss (MD -21.65, P = 0.02) and fewer lymph nodes harvested (MD 6.23, P<0.001). Furthermore, the number of metastatic lymph nodes was similar between the two groups (MD 0.62, P = 0.07), with the exception of lymph node stations 5 and 6, where the metastatic rates in the TG group were 0.82% and 1.6% (P = 0.645), respectively. Postoperative complications were lower in the PG group, but the difference was not statistically significant (OR 1.24, P = 0.289). Hospital stay was significantly shorter in the PG group (MD 0.81, P = 0.001). No significant differences in the 5-year OS or RFS were found (HR 0.99, P = 0.48 for OS; HR 0.83, P = 0.87 for RFS). Sensitivity and publication bias analyses supported the robustness and consistency of the results.

**Conclusion:**

For selected patients with LAPGC, PG offers similar curative potential and oncological efficacy as TG, making it a safe option.

## Introduction

Proximal gastric cancer (PGC), which refers to tumors located in the upper third of the stomach, has been increasing in incidence globally. This trend is especially notable in East Asia and Western countries, possibly due to changes in diet, rising obesity rates, and widespread eradication of *Helicobacter pylori* (1–3). Advancements in early detection and the growing use of neoadjuvant therapy have led to an increasing number of patients being diagnosed with locally advanced PGC (LAPGC). For these patients, surgery remains the primary curative treatment (4, 5). Traditionally, total gastrectomy (TG) has been the preferred approach for LAPGC due to concerns about achieving complete oncologic resection, performing extensive lymphadenectomy, and avoiding proximal margin involvement (6, 7). However, TG has several well-known postoperative drawbacks, such as severe nutritional impairment, loss of the gastric reservoir and digestive hormones, and long-term reliance on nutritional supplements. These complications can significantly reduce quality of life and survival, particularly in elderly or nutritionally vulnerable patients (3, 8–10).

Proximal gastrectomy (PG), initially used only for early-stage PGC, has recently regained attention as a function-preserving alternative to total gastrectomy (TG). This renewed interest is largely due to the development of advanced reconstruction techniques, including double-tract reconstruction, jejunal interposition, and side overlap anastomosis (9, 11, 12). These techniques are designed to reduce reflux esophagitis and anastomotic complications, which were major concerns in earlier surgical practices. The main advantage of PG is the preservation of the distal stomach and pyloric function. This allows for improved food intake, better absorption of micronutrients (especially iron and vitamin B12), and a lower risk of sarcopenia and hypoalbuminemia (9). In patients with LAPGC who respond well to neoadjuvant chemotherapy, PG combined with adequate lymphadenectomy may provide oncologic outcomes comparable to TG while preserving better postoperative function (8, 13, 14).

Despite these theoretical advantages, PG remains underutilized for LAPGC due to persistent doubts regarding its oncologic safety (15). Concerns include potentially inadequate dissection of lymph node stations 5 and 6, increased risk of locoregional recurrence, and technical challenges related to anastomotic reconstruction (14). Furthermore, the lack of randomized controlled trials (RCTs) has limited the generalizability of available evidence. Most existing data come from retrospective observational studies, many of which are subject to confounding by indication and selection bias (16, 17). However, the recent proliferation of well-designed propensity score-matched (PSM) studies has helped mitigate these limitations by balancing baseline characteristics between groups, thereby enhancing the validity of comparative outcomes (1, 8, 13, 18, 19).

Given the emergence of PSM studies comparing PG and TG for LAPGC, there is now an opportunity to synthesize the available evidence in a comprehensive and statistically robust manner. Unlike previous meta-analyses that combined unmatched or early-stage gastric cancer populations, our study uniquely focuses on PSM cohorts specifically in the setting of proximal advanced gastric cancer. This approach helps reduce confounding and provides a more targeted and reliable comparison of surgical strategies in this clinically challenging subgroup. In this study, we conducted a meta-analysis of PSM studies comparing PG and TG in patients with LAPGC, aiming to evaluate whether PG can achieve equivalent oncologic outcomes while preserving postoperative function and reducing complications. Our primary endpoints were overall survival (OS) and recurrence-free survival (RFS), while secondary outcomes included surgical outcomes and postoperative complications. The findings aim to support surgical decision-making in this increasingly relevant clinical context.

## Materials and methods

This systematic review and meta-analysis was performed and reported following the Preferred Reporting Items for Systematic Reviews and Meta-analysis (PRISMA) Statement (20) and AMSTAR-2 (Assessing the methodological quality of systematic reviews) (21) guidelines (Supplemental materials).

### Search strategy

A comprehensive literature search was performed across PubMed, EMBASE, and the Cochrane Library, covering studies published up to December 31, 2024. The search strategy used a combination of the following terms: (“proximal gastric cancer” OR “upper third gastric cancer” OR “adenocarcinoma of esophagogastric junction” OR “AEG” OR “advanced”) AND (“proximal gastrectomy”). The full search strategy is provided in **Supplementary Table 1**. To focus on contemporary evidence, the inclusion criteria were restricted to articles published between January 1, 2015, and December 31, 2024. Only the studies published in English were considered. Manual cross-checking of the reference lists in the retrieved articles was performed to identify additional relevant studies. References were managed using EndNote software (version X9; Clarivate), and duplicates were manually excluded. As this analysis exclusively utilized publicly available data, ethical approval and patient consent were not required.

### Study selection

Two researchers independently evaluated the retrieved studies. Initial screening of titles and abstracts excluded non-relevant publications, including case reports, letters, reviews, and unrelated articles. Subsequently, the full-text articles were rigorously evaluated to confirm alignment with the inclusion criteria. Guided by the PICOS framework (population, intervention, comparator, outcome, study design) (22), eligibility criteria were defined as follows: population—patients diagnosed with LAPGC; intervention—PG versus TG; outcomes—reported at least one of the following outcomes: OS, RFS, and surgical outcomes; study design—RCTs or PSM studies. The exclusion criteria were as follows: (a) reviews, conference abstracts, commentaries, letters, or animal studies; (b) non-English publications; (c) pathological diagnoses of non-target malignancies (e.g., gastrointestinal stromal tumors); and (d) studies involving early-stage disease only or salvage surgery. Two authors independently screened titles, abstracts, and full texts, with disagreements resolved by consensus or a third reviewer.

### Data extraction

Data were extracted independently by two investigators using a standardized form, including: (1) study characteristics (first author’s surname, publication year, study design, country, cohort size); (2) cohort demographics (mean age and sex distribution); (3) surgical metrics (operative duration, intraoperative blood loss, dissected lymph nodes); (4) postoperative complications (early complications ≤30 days, Clavien-Dindo grade); and (5) 5-year OS and/or 5-year RFS, recurrence patterns, and adjuvant chemotherapy. A third independent reviewer re-evaluated all data, with discrepancies resolved through consensus discussions to minimize bias and ensure reliability.

### Quality assessment

Quality of included studies was assessed using the Risk of Bias in Non-randomized Studies of Interventions (ROBINS-I) tool (23), which evaluates bias across seven domains: confounding, participant selection, intervention classification, deviations from intended protocols, missing data, outcome measurement, and selective reporting. Each domain was rated as “Low,” “Moderate,” “Serious,” “Critical,” or “No information,” with the highest risk level across domains determining the overall bias classification for each study. Two reviewers independently assessed study quality according to the ROBINS-I guidelines, resolving discrepancies through arbitration by a third author.

### Statistical analysis

Pooled hazard ratios (HRs) for OS and DFS and odds ratios (ORs) for dichotomous outcomes were calculated using a random-effects model (DerSimonian–Laird method) to account for between-study heterogeneity. If HRs were not directly reported, they were estimated from Kaplan–Meier curves using the Tierney method. Weighted mean differences (MDs) were calculated for continuous variables. Heterogeneity was quantified using Cochran’s Q test and I² statistics, with I² thresholds defined as follows: <25% (negligible), 25–50% (moderate), and >50% (substantial). Forest plots visualized study-specific and aggregated results, whereas subgroup analyses explored sources of heterogeneity. Publication bias was evaluated using funnel plots. Sensitivity analyses (leave-one-out method) were performed for oncological outcomes (OS/RFS) to evaluate robustness by iteratively excluding individual studies. All statistical analyses were performed using Review Manager (RevMan) version 5.4 and R (v4.3.2)/RStudio (v4.2.2). A p-value <0.05 was considered statistically significant.

## Results

### Study selection and characteristics

**Figure 1:**
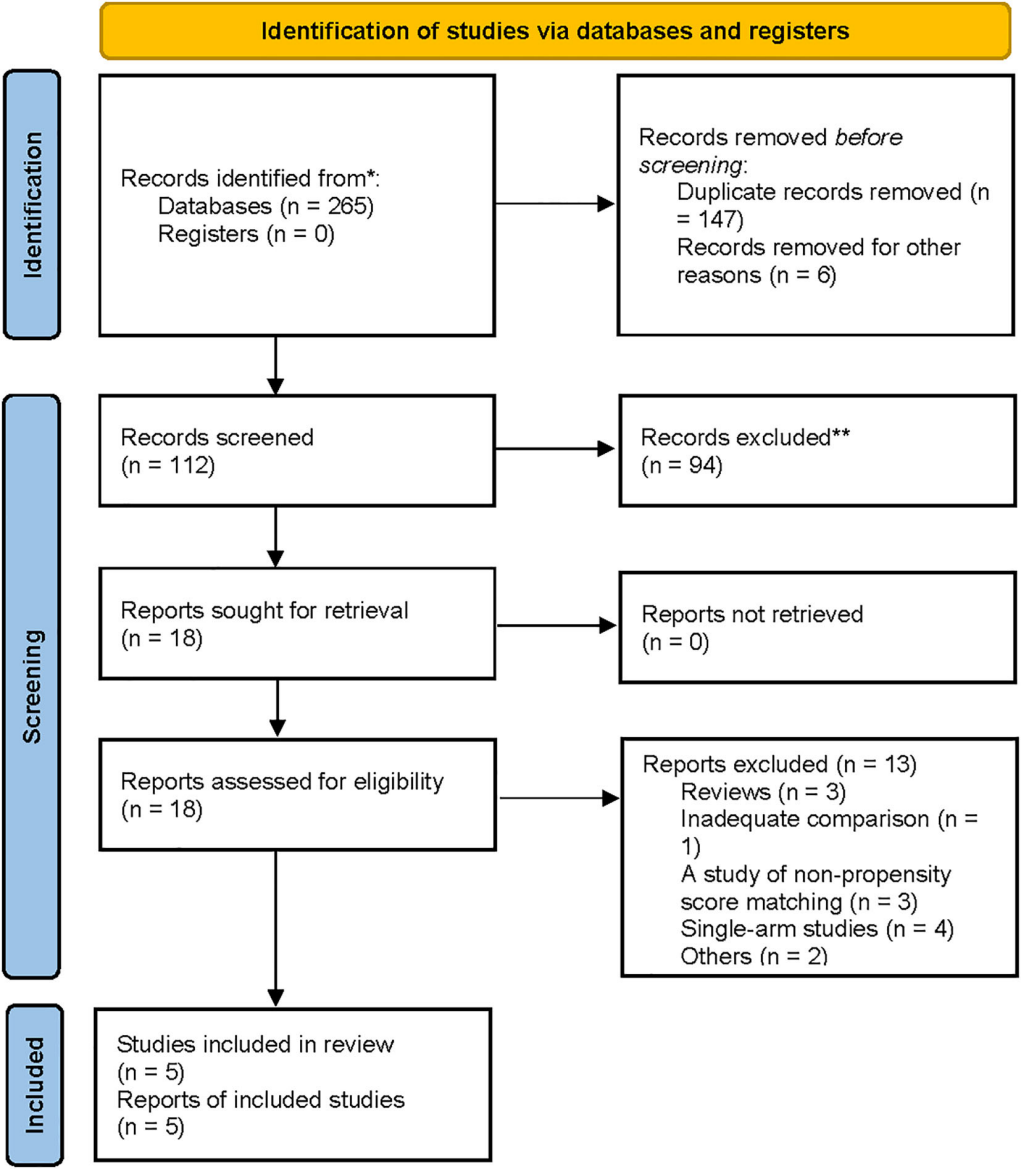
Flow chart of the search for eligible studies.

A flowchart outlining the process and results of study selection is shown in **Figure 1**. A total of 265 articles were identified through searches in the PubMed, Embase, and Cochrane Library databases. After screening titles and abstracts, and removing duplicates and irrelevant studies, 18 studies remained for full-text review according to the inclusion and exclusion criteria. Finally, five studies were included in this meta-analysis (1, 8, 13, 18, 19), all of which had a retrospective design (**Tables 1**, **2**). All studies were published between 2020 and 2024. The sample size of each study ranged from 274 to 2918. After propensity score matching, 412 patients were included in both the PG and TG groups. The initial agreement between the two investigators regarding study selection was high (κ = 0.92). Any discrepancies were resolved by consensus with a third reviewer.

**Table 1:** Characteristics of included studies.

First author	Year	Study period	Study design	No. patients		Inclusion criteria
				Before PSM	After PSM	
Zhao et al. (19)	2020	1998-2018	Multi-Center Retrospective PSM Cohort Study	2918	300	LAPGC, which was defined as clinical stage IB–III (according to the eighth edition of the International Union against Cancer Classification) with the epicenter located in cardia or fundus.
Lee et al. (1)	2024	2007-2018	Single-Center Retrospective PSM Cohort Study	713	110	Patients with advanced gastric cancer at the upper-third level of the stomach or EGJ cancer underwent curative PG or TG with standard LN dissection.
Gu et al. (13)	2024	2009-2022	Single-Center Retrospective PSM Cohort Study	330	120	Patients underwent TG or PG after neoadjuvant therapy for histologically confirmed locally advanced gastric cancer.
Peng et al. (18)	2021	2011-2017	Single-Center Retrospective PSM Cohort Study	329	134	(1) age range: 25-85 years; (2) pathologically diagnosed with adenocarcinoma; (3) tumors were located in the upper third of the stomach; (4) Pathological T stage: T2 to T4 (advanced gastric cancer); (5) undergoing TG or PG.
Yuan et al. (8)	2023	2009-2022	Multi-Center Retrospective PSM Cohort Study	274	160	(1) patients diagnosed with locally advanced GC with tumors located in the upper third of the stomach without esophagogastric junction adenocarcinomas; (2) tumor stage ranging from TNM stage I to III, with no evidence of distant metastases; (3) patients who underwent at least NACT followed by minimally invasive radical gastrectomy (laparoscopic or robotic surgery); (4) American Society of Anesthesiology score of class I, II, or III.

LAPGC, locally advanced proximal gastric cancer; PG, proximal gastrectomy; PSM, propensity score-matched; TG, total gastrectomy.

**Table 2:** Baseline demographics of the included studies after PSM.

First author	Age (years)	Gender (male)	BMI	ASA score (1-2/3)	TRG (0/1/2/3)	Pathological TNM stage			Neoadjuvant therapy	Operative approach		
						Ib	II	III		Open	Laparoscopic	Robotic
Zhao et al. (19)	TG: >65 (47) PG: >65 (47)	TG: 127 PG: 127	TG: 23.8±3.4 PG: 23.8±3.5	NA	NA	TG: 9 PG: 8	TG: 59 PG: 49	TG: 82 PG: 93	TG: 75 PG: 65	TG: 104 PG: 146	TG: 39 PG: 2	NA
Lee et al. (1)	TG: 61.8 ± 11.0 PG: 62.2 ± 12.3	TG: 60 PG: 34	NA	NA	NA	NA	NA	NA	NA	TG: 42 PG: 42	TG: 14 PG: 18	TG: 4 PG: 0
Gu et al. (13)	TG: 65 (61–69) PG: 63 (58–66)	TG: 43 PG: 41	NA	TG: 66/5 PG: 36/3	TG: 5/10/37/19 PG: 5/5/22/7	TG: 25 PG: 21	TG: 28 PG: 28	TG: 7 PG: 11	TG: 71 PG: 39	TG: 54 PG: 29	TG: 17 PG: 10	NA
Peng et al. (18)	TG: 64.6 ± 6.8 PG: 64.3 ± 8.0	TG: 48 PG: 48	NA	NA	NA	TG: 19 PG: 17	TG: 31 PG: 30	TG: 17 PG: 20	NA	NA	NA	NA
Yuan et al. (8)	TG: 64 (58-68) PG: 63 (58-68)	TG: 68 PG: 70	TG: 22.9 (21.1-25.3) PG: 23.1 (21.1-25.9)	TG: 68/12 PG: 69/11	TG: 5/15/29/31 PG: 6/14/33/27	TG: 25 PG: 31	TG: 28 PG: 26	TG: 27 PG: 23	TG: 80 PG: 80	NA	TG: 64 PG: 58	TG: 16 PG: 22

ASA, American society of Aneshesiologists; BMI, body mass index; NA, not available; PG, proximal gastrectomy; PSM, propensity score-matched; TG, total gastrectomy; TRG, tumor regression grade.

### Quality assessment

**Figure 2:**
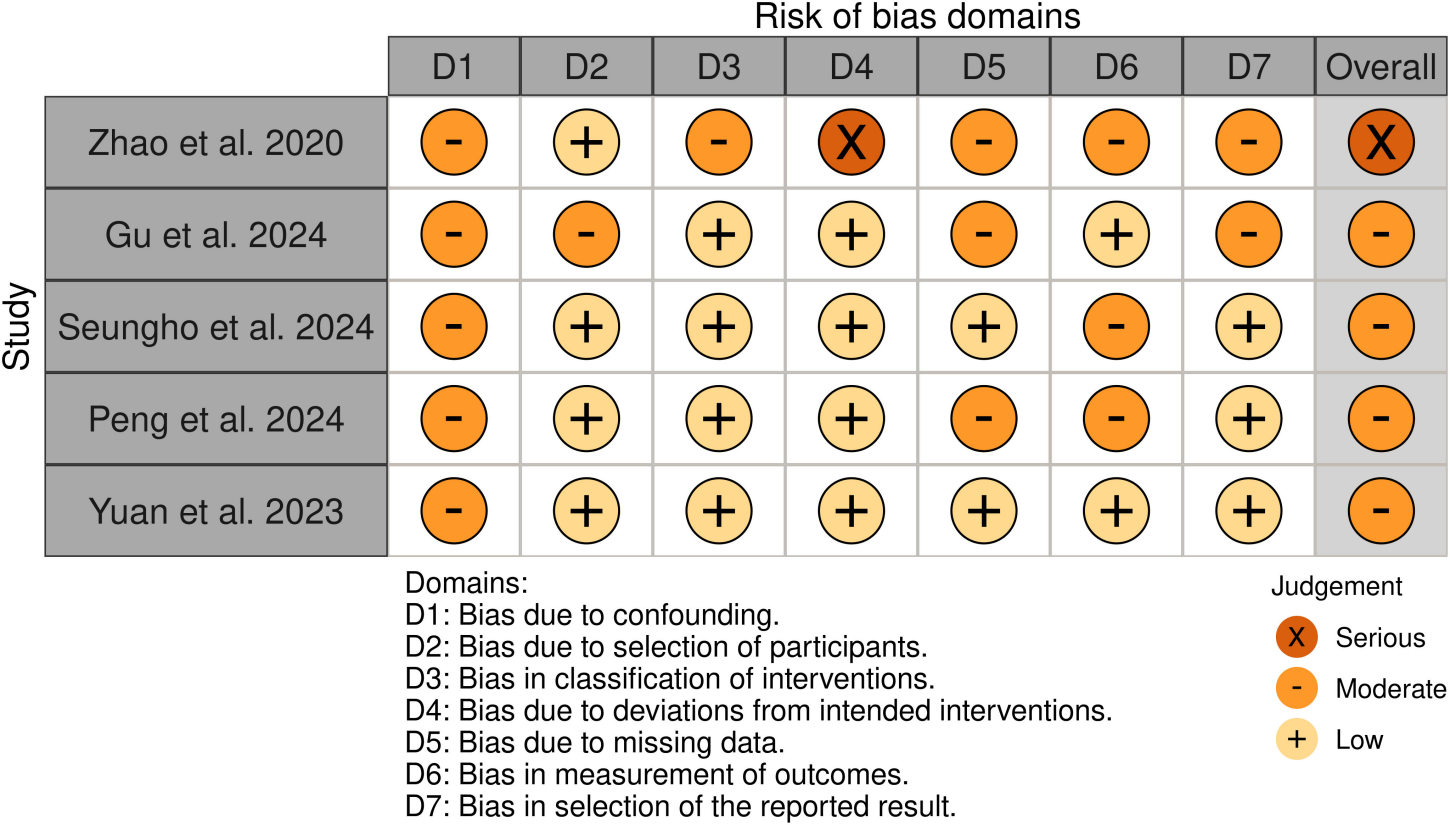
Risk of bias plot following the ROBINS- I tool for quality assessment.

Among the 5 non-randomized studies included in our systematic review, the methodological quality assessment using the ROBINS-I tool revealed the following distribution: four studies were assessed as having a “ Moderate “ risk of bias (1, 8, 13, 18), and one study demonstrated a “ Serious” risk of bias (19). The primary methodological limitations contributing to the elevated risk of bias were the absence of appropriate control groups and potential presence of unmeasured confounders, which may have influenced the observed outcomes. The comprehensive risk of bias assessment for each individual study is presented in **Figure 2**, providing a detailed overview of the methodological quality across all the included studies.

### Surgical and perioperative outcomes

**Figure 3:**
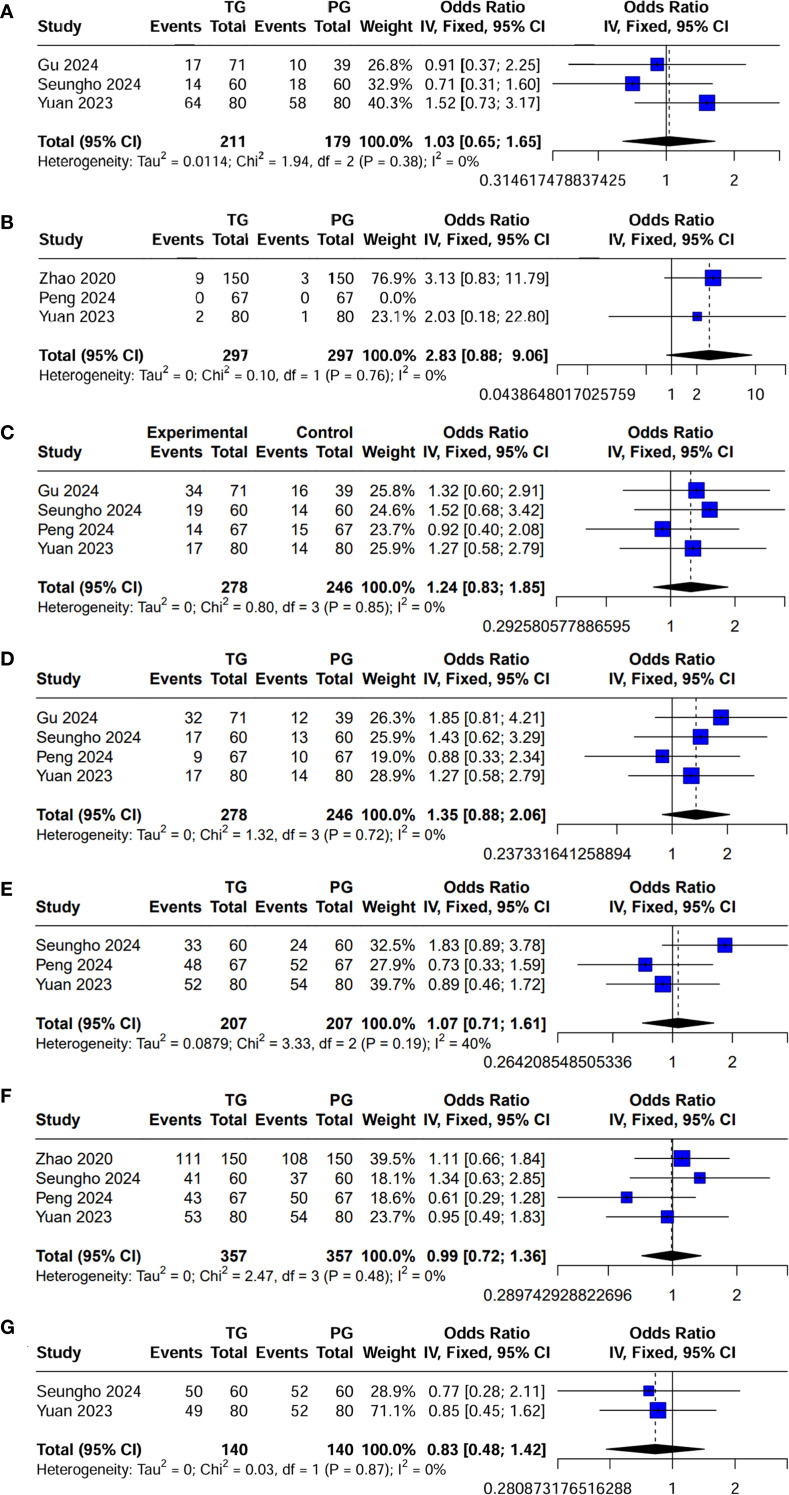
Forest plot of dichotomous outcomes. **(A)** Laparoscopic surgery rates; **(B)** Positive surgical margins; **(C)** Postoperative complications; **(D)** Postoperative complications classified as Clavien-Dindo grade ≥ II; **(E)** Adjuvant chemotherapy administration; and **(F)** 5-year OS; and **(G)** 5-year RFS.

**Figure 4:**
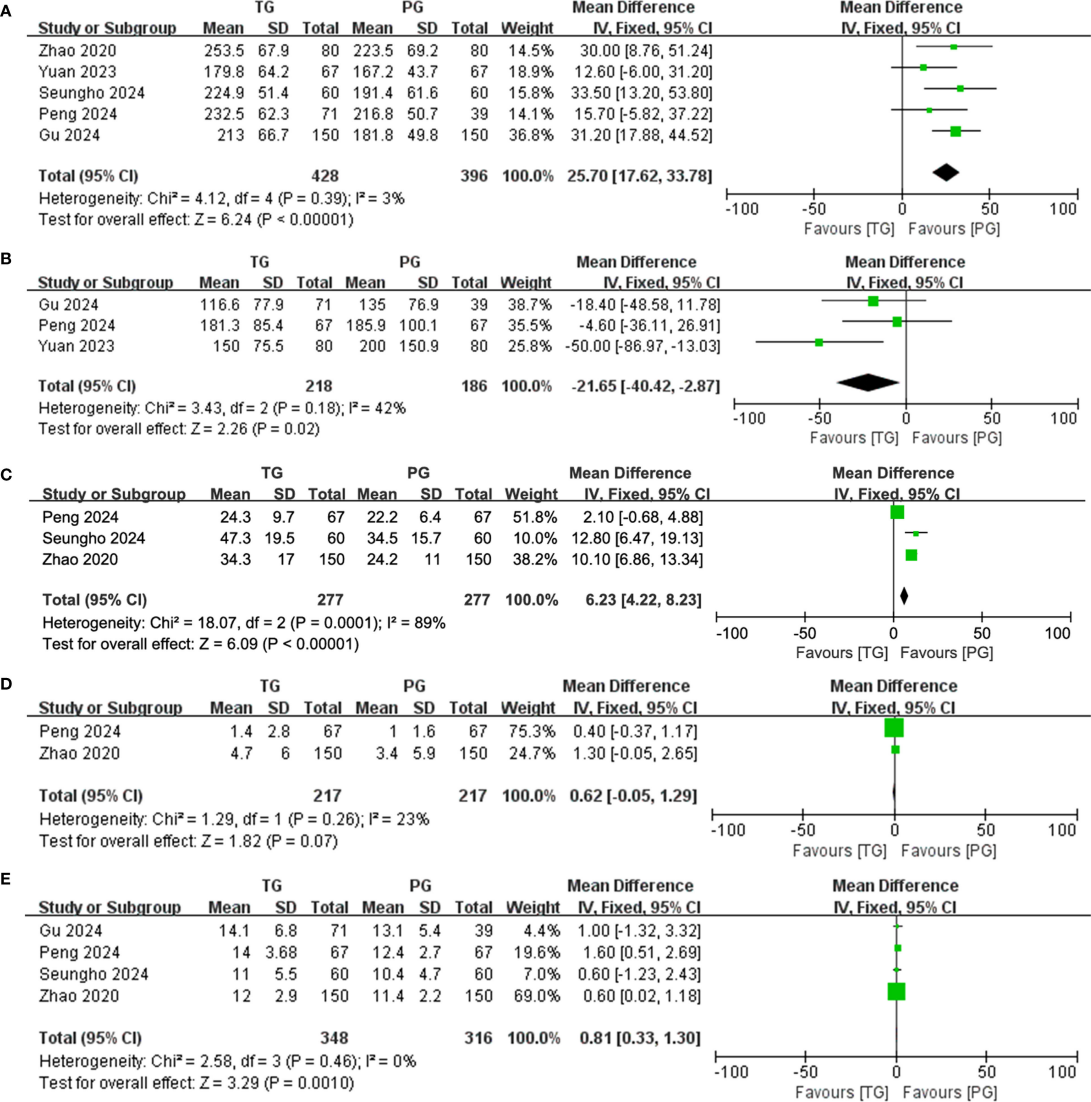
Forest plot of continuous variables. **(A)** Operative time; **(B)** Blood loss; **(C)** Number of lymph nodes harvested; **(D)** Number of metastatic lymph nodes; and **(E)** hospital stay.

The surgical and perioperative outcomes are presented in **Table 3**. The laparoscopic surgery rates were comparable between the PG and TG groups [OR (95%CI) 1.03 (0.65, 1.65), I²=0%, P = 0.896] (**Figure 3A**). However, the PG group had significantly shorter operative times [MD (95%CI) 25.7 (17.62, 33.78), I²=3%, P<0.001] (**Figure 4A**). Negative values for blood loss indicate that the PG group had lower estimated blood loss compared to the TG group [MD (95%CI) -21.65 (-40.42, -2.87), I²=42%, P = 0.02] (**Figure 4B**). In terms of lymph node dissection, the PG group had a lower number of lymph nodes harvested compared to the TG group [MD (95%CI) 6.23 (4.22, 8.23), I²=89%, P<0.001] (**Figure 4C**). The heterogeneity observed in both the regional and minimally invasive surgery proportion (>50% vs. <50%) subgroup analyses appear to be primarily driven by the study by Peng 2024, with a lesser contribution from Zhao 2020 (**Supplementary Figures 1A, B**). These inconsistencies may be attributed to the learning curve associated with minimally invasive techniques or variations in surgeon experience across institutions. Furthermore, the number of metastatic lymph nodes was similar between the two groups [MD (95%CI) 0.62 (-0.05, 1.29), I²=23%, P = 0.07] (**Figure 4D**), with the exception of lymph node stations 5 and 6, where the metastatic rates in the TG group were 0.82% (I²=0%, P = 0.746) and 1.6% (I²=0%, P = 0.645), respectively. This suggests that while PG involves fewer lymph nodes dissected, the oncologic outcomes in terms of lymph node metastasis were comparable. The rate of positive surgical margins was lower in the PG group [OR (95%CI) 2.83 (0.88, 9.06), I²=0%], although the difference did not reach statistical significance (P = 0.08) (**Figure 3B**).

**Table 3:** Surgical characteristics of TG vs. PG for LAPGC after PSM.

First author	Operative time, min*	Blood loss, ml*	Dissected lymph nodes*	No. of lymph nodes metastasis	Frequency of NO.5 or NO.6 LN metastasis after TG.	Positive surgical margin
Zhao et al. (19)	TG: 213.5±66.7 PG: 181.8±49.8	NA	TG: 34.3±17.0 PG: 24.2±11.0	TG: 3.4+5.9 PG: 4.7+6.0	TG: 9 (6.00) PG: 3 (2.00)	TG: 9 (6.00) PG: 3 (2.00)
Gu et al. (13)	TG: 216.8+50.7 PG: 232.5+62.3	TG: 116.6+77.9 PG: 135+76.9	NA	NA	NA	NA
Lee et al. (1)	TG: 224.9 ± 51.4 PG: 191.4 ± 61.6	NA	TG: 47.3+19.5 PG: 34.5+15.7	NA	NA	NA
Peng et al. (18)	TG: 179.76 ± 64.15 PG: 167.15 ± 43.73	TG: 181.34 ± 85.44 PG: 185.97 ± 100.09	TG: 24.34 ± 9.66 PG: 22.19 ± 6.38	TG: 1.42 ± 2.81 PG: 1.00 ± 1.60	TG: 0 PG: 0	TG: 0 PG: 0
Yuan et al. (8)	TG: 253.5+67.9 PG: 223.5+69.2	TG: 150+75.5 PG: 200+150.9	NA	NA	TG: 2 (2.5) PG: 1 (1.3)	TG: 2 (2.5) PG: 1 (1.3)

*Operative time/Blood loss/Anastomotic time/Dissected lymph nodes: mean (standard deviation).

NA, not available.

### Postoperative complications and hospital stay

Postoperative complications and hospital stay are detailed in **Table 4**. Regarding postoperative complications, the overall incidence was lower in the PG group than in the TG group [OR (95%CI) 1.24 (0.83, 1.85), I²=0%], though this difference was not statistically significant (P = 0.289) (**Figure 3C**). Subgroup analysis further revealed that the incidence of complications classified as Clavien-Dindo grade ≥ II was also lower in the PG group [OR (95%CI) 1.35 (0.88, 2.06), I²=0%], with no statistical significance either (P = 0.165) (**Figure 3D**). This could imply that while PG may reduce certain severe complications, the effect may not be sufficiently large to achieve statistical significance in a propensity-matched cohort. A significant finding was the hospital stay, where patients in the PG group had a notably shorter recovery time [MD (95%CI) 0.81 (0.33, 1.30), I²=0%, P = 0.001] (**Figure 4E**). The rate of adjuvant chemotherapy administration was similar between the two groups [OR (95%CI) 1.07 (0.71, 1.61), I²=40%, P = 0.19] (**Figure 3E**), suggesting that both groups were equally likely to receive further oncologic treatment postoperatively, reflecting similar staging and treatment protocols.

**Table 4:** Short- and long-term outcomes of TG vs. PG for LAPGC after PSM.

First author, year	Early complications, n (%)					Hospital stays (d)	Adjuvant chemotherapy, n (%)	5-year OS	5-year RFS
	Overall	CD grade I	CD grade II	CD grade III	CD grade IV				
Zhao et al. (19)	NA	NA	NA	NA	NA	TG: 12 [10–14] PG: 11 [10–13]	NA	TG: 74.5% PG: 72.0%	NA
Gu et al. (13)	TG: 34 (47.9) PG: 16 (41.0)	TG: 2 (2.8%) PG: 1 (2.6%)	TG: 20 (28.2) PG: 9 (23.1)	TG: 11 (15.5) PG: 3 (7.7)	TG: 1 (1.4) PG: 3 (7.7)	TG: 13 (10–19) PG: 12 (10–17)	NA	P = 0.81	NA
Lee et al. (1)	TG: 19 (26.8) PG: 14 (35.9)	TG: 2 (3.3) PG: 1 (1.7)	TG: 13 (21.7) PG: 11 (18.3)	TG: 4 (6.7) PG: 1 (1.7)	TG: 0 (0) PG: 1 (1.7)	TG: 11.0 ± 5.5 PG: 10.4 ± 4.7	TG: 33 (55.0) PG: 24 (60.0)	TG: 68.3% PG: 61.7%	TG: 83.3% PG: 86.7%
Peng et al. (18)	TG: 14 (20.9) PG: 15 (22.4)	TG: 6 (9.0) PG: 7 (10.4)	TG: 4 (6.0) PG: 5 (7.5)	TG: 5 (7.5) PG: 4 (6.0)	TG: 0 (0) PG: 1 (1.5)	TG: 14.00 ± 3.68 PG: 12.43 ± 2.70	TG: 48(71.6) PG: 52(77.7)	TG: 64.3% PG: 74.9%	NA
Yuan et al. (19)	TG: 17 (21.3) PG: 14 (17.5)	NA	TG: 17 (21.3) PG: 14 (17.5)	NA	NA	NA	TG: 52 (65.0) PG: 54 (67.5)	TG: 66.0% PG: 68.4%	TG: 61.9% PG: 64.8%

CD, Clavien-Dindo; LAPGC, locally advanced proximal gastric cancer; PG, proximal gastrectomy; PSM, propensity score-matched; OS, overall survival; RFS, recurrence-free survival; TG, total gastrectomy.

### Oncological outcomes

There was no significant difference in the 5-year OS between the PG and TG groups [HR (95%CI) 0.99 (0.72, 1.36), I²=0%, P = 0.48] or in the 5-year RFS [HR (95%CI) 0.83 (0.48, 1.42), I²=0%, P = 0.87] (**Figures 3F, G**; **Table 4**). These findings indicate that despite differences in surgical approach, both procedures yield comparable long-term survival outcomes, highlighting the feasibility of PG as an oncological safe alternative to TG for locally advanced proximal gastric cancer.

### Publication bias for 5-year OS and RFS

**Figure 5:**
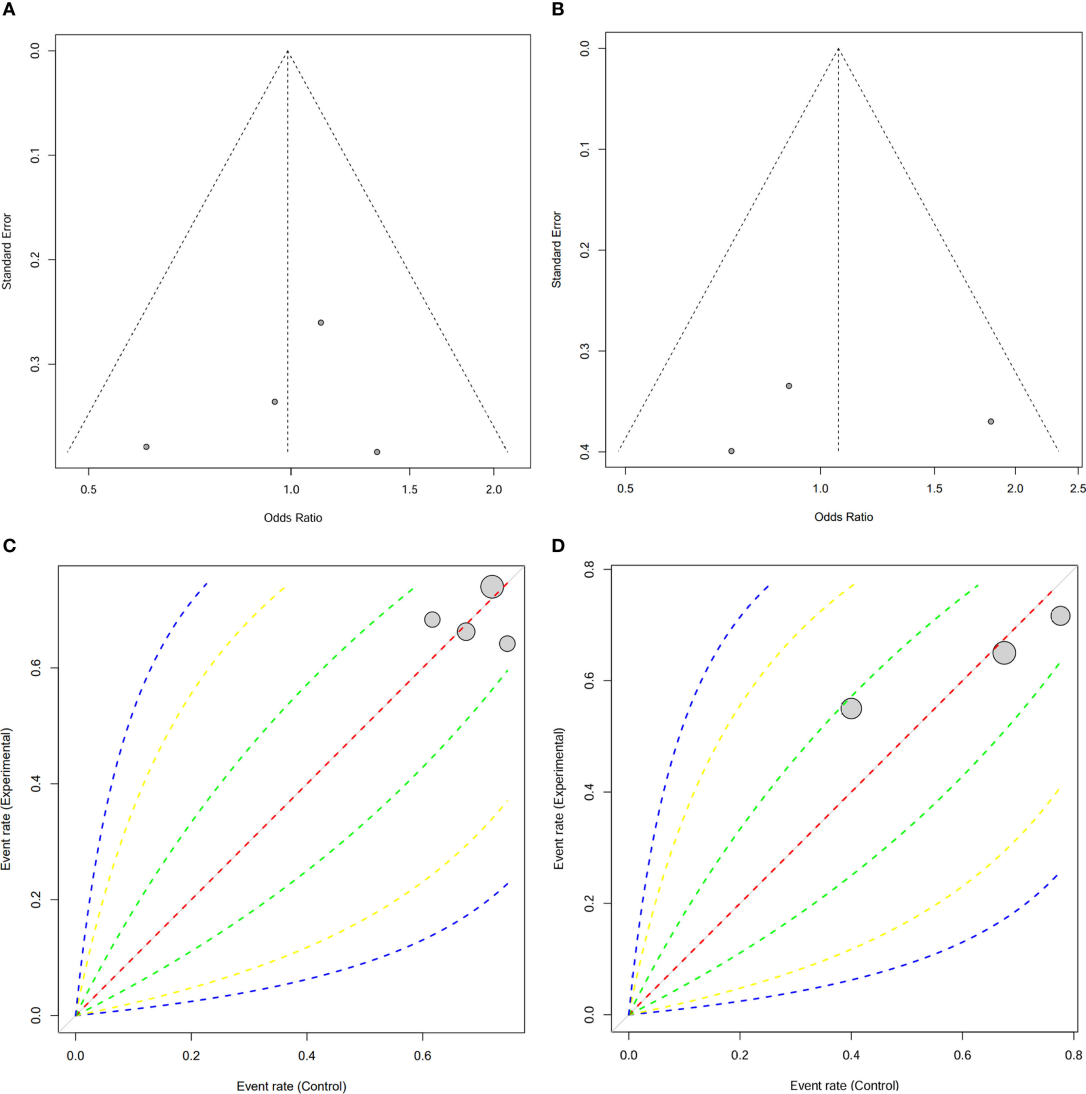
Publication bias assessment for 5-year OS and 5-year RFS. **(A)** Funnel plot for 5-year OS and **(B)** 5-Year RFS; **(C)** Labbe plot for 5-year OS and **(D)** 5-Year RFS.

To assess the robustness of the results, funnel plots for both OS and RFS were generated. The funnel plots showed no significant asymmetry, indicating the absence of publication bias (**Figures 5A, B**). In addition, Labbe plots confirmed that all studies were closely distributed around the reference line, further suggesting minimal heterogeneity across the included studies (**Figures 5C, D**). These analyses reinforce the reliability and consistency of our meta-analysis findings.

### Sensitivity analysis for 5-year OS and RFS

**Figure 6:**
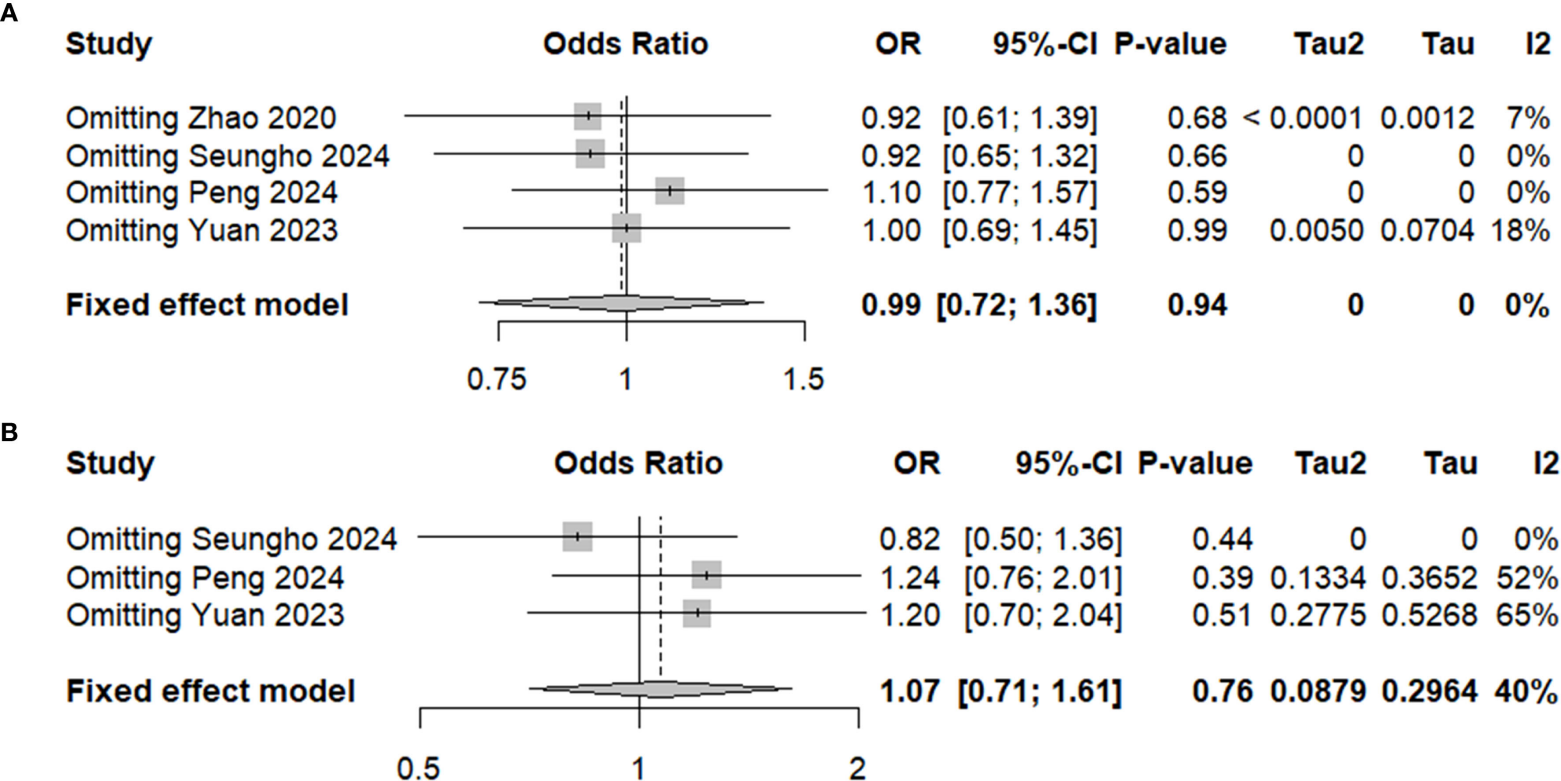
Sensitivity analysis for **(A)**5-Year OS and **(B)** 5-Year RFS.

The sensitivity analysis for both 5-year OS and RFS showed that excluding any single study did not significantly alter the pooled effect sizes, indicating the robustness of the results (**Figures 6A, B**). For both OS and RFS, the summary effect remained consistent, suggesting that neither PG nor TG had a considerable impact on long-term survival outcomes. These findings underscore the reliability of our conclusion that PG is comparable to TG in terms of both 5-year OS and RFS for patients with locally advanced proximal gastric cancer.

## Discussion

The comparison between PG and TG for treating LAPGC has long attracted attention in surgical oncology. This systematic review and meta-analysis offers a comprehensive evaluation of PG versus TG, focusing on propensity score-matched studies and comparing surgical, perioperative, and oncological outcomes. The results suggest that PG and TG yield comparable long-term survival, despite differences in surgical techniques, postoperative complications, and recovery times.

One key finding of this meta-analysis was that PG resulted in significantly shorter operative times than TG, with an average reduction of approximately 25.7 minutes. This finding is consistent with previous studies suggesting that PG, which involves resection of the proximal stomach while preserving the pylorus, is technically less demanding and therefore requires less operative time. However, PG was associated with significantly greater intraoperative blood loss than TG (P = 0.02), possibly due to more complex dissection around the cardia and greater curvature, which are areas rich in blood vessels. Despite the increased blood loss, the overall surgical complexity and risk profile of PG remain acceptable. The reduced operative time may be particularly beneficial for elderly or frail patients.

Postoperative complications, which often lead to prolonged hospital stays and increased healthcare costs, are critical indicators when assessing the safety and technical feasibility of surgical procedures (9, 24, 25). Although previous studies have reported a higher incidence of anastomotic stenosis and reflux esophagitis after PG, these complications are largely influenced by the type of reconstruction technique used (3, 26–28). This study did not evaluate reflux esophagitis, as its occurrence is well known to depend on the specific reconstruction technique used in PG. For other postoperative complications, available data in the literature remain relatively limited. In this study, the overall rate of postoperative complications was slightly lower in the PG group than in the TG group; however, the difference was not statistically significant. This suggests that although PG may help reduce the risk of certain complications, the benefit may be insufficient to achieve a statistically significant difference in a propensity score-matched cohort. Moreover, the incidence of severe complications (Clavien–Dindo grade≥II) was also lower in the PG group, possibly reflecting the less invasive nature of the procedure. Another notable finding was a significant reduction in hospital stay among PG patients, with an average decrease of 0.81 days. This aligns with the general view that PG, as a less extensive procedure, promotes faster recovery and shorter hospital stays (9, 28, 29). Shorter hospital stays benefit patients by lowering the risk of hospital-acquired infections and reducing overall healthcare costs (30). Faster recovery associated with PG may be especially beneficial for elderly patients or those with multiple comorbidities, who are more prone to delayed recovery after major surgeries such as TG.

Oncological safety should remain the primary consideration when choosing between PG and TG (17). TG is a more extensive procedure that ensures complete resection of potential residual tumor at the gastric margin, often resulting in a lower recurrence rate than PG (1). However, previous studies have reported no significant difference in survival outcomes between PG and TG for early-stage proximal gastric cancer (1, 9, 28), which aligns with our findings in LAPGC. Khalayleh et al. further recommended PG for differentiated cT1–T3N0/N1 or poorly differentiated cT1N0/N1 gastric cancers smaller than 4 cm (31). Beyond tumor size, the extent of lymph node metastasis is widely recognized as a key prognostic factor for survival outcomes (32–34). Subgroup analyses of OS by ypN stage have shown that higher ypN stages are associated with worse survival, although multivariate analysis revealed no significant differences (13). Evaluating lymph node metastasis rates at stations #4d, #5, #6, and #12a—which are typically dissected in TG but not in PG—can help clarify this concern (35). Yura et al. (36) reported minimal metastasis rates at these stations in patients with T2/T3 proximal gastric cancer: 0.99%, 0%, 0%, and 0.006%, respectively. Similarly, Sasako et al. (37) found that stations #5 and #6 had the lowest therapeutic indices in upper-third tumors compared to those in the middle or lower third. Ooki et al. (38) reported nodal metastasis rates of 3.7%, 2.4%, and 0% at stations #4d, #5, and #6 in T3 proximal cancers. Similarly, Haruta et al. (39) found rates of 3.3%, 0.5%, 1.6%, and 0% at the same stations in proximal cancers. According to Niihara et al. (40), in early proximal gastric cancer, lymph nodes along the left gastric artery (#1, #3, and #7) serve as primary drainage sites, while those along the right gastric artery (#5, #8, and #12) and right gastroepiploic arteries (#4d and #6) are more distant and rarely involved in early metastasis. These findings suggest that prophylactic distal lymphadenectomy may have limited clinical value. Moreover, a recent multivariate analysis by Yang et al. (41) revealed that tumors ≥4 cm and metastases to stations #4, #7, #8, or #9 are independent risk factors for involvement of stations #5 and/or #6. Since these distal stations are typically excluded in PG, such findings underscore the potential risk of residual nodal disease when PG is applied to patients with high-risk profiles. Thus, in cases of advanced proximal tumors with suspected extended nodal spread, TG with standard D2 lymphadenectomy may remain the more appropriate oncologic option. Lymph node dissection was another important aspect evaluated in this meta-analysis. PG was associated with a significantly lower number of harvested lymph nodes compared to TG. This is expected, as TG generally includes more extensive lymphadenectomy, particularly in the perigastric and para-aortic regions. Nevertheless, despite fewer lymph nodes being dissected in PG, the metastatic rates were comparable between the two groups, suggesting that PG does not compromise oncologic outcomes related to lymph node involvement. These findings are consistent with previous studies showing that limited lymphadenectomy in PG may still provide sufficient oncologic control, especially when combined with adjuvant therapy (42). In this context, it is important to acknowledge the controversial role of extended lymphadenectomy, particularly para-aortic lymph node dissection (PAND), in the treatment of LAPGC following neoadjuvant chemotherapy. Although PAND is not routinely recommended, selective studies have reported that it may offer survival benefit in patients with radiologically suspected para-aortic node metastases who respond well to preoperative chemotherapy. However, given the technical demands, increased perioperative morbidity, and limited evidence supporting routine use, PAND is rarely performed, especially in conjunction with proximal gastrectomy. Its feasibility is mostly confined to highly selected patients in high-volume centers with experienced surgical teams (43). Adjuvant chemotherapy has been proven to improve survival, particularly in advanced gastric cancer, by targeting micrometastases and lowering recurrence risk. This highlights the importance of integrating surgical resection with chemotherapy to optimize long-term outcomes, regardless of the surgical approach.

Despite the differences in surgical techniques and perioperative outcomes, both PG and TG demonstrated comparable long-term oncologic outcomes, as reflected in 5-year OS and RFS. Although PG entails a less extensive resection, it can achieve oncologic results equivalent to those of TG in patients with LAPGC. Several factors may explain the lack of significant differences in survival outcomes. First, the high rate of adjuvant chemotherapy administration—without significant difference between the PG and TG groups—indicates that postoperative treatment protocols were largely comparable, potentially offsetting any differences in surgical extent. Second, although PG involves more limited lymphadenectomy, the similar rates of metastatic lymph nodes in both groups suggest that oncologic control is not substantially compromised. Furthermore, the established role of chemotherapy in managing advanced gastric cancer likely mitigates the potential drawbacks of less extensive surgery. Sensitivity analyses confirmed the robustness of these findings, showing no material change in results upon exclusion of any single study. This enhances the reliability of the conclusion that PG is not inferior to TG in terms of survival outcomes. Additionally, the absence of significant publication bias, as demonstrated by funnel and L’Abbé plots, further reinforces the validity of the results and suggests minimal influence from selective reporting or unpublished data.

Quality of life (QoL) has been an important factor in comparing PG and TG, particularly in early-stage proximal gastric cancer. Most studies suggest that PG is more beneficial in maintaining nutritional status, as it is a function-preserving procedure that leads to less postoperative weight loss and better overall nutrition, primarily due to the preservation of the gastric fundic gland region (9, 11, 44). However, there is a lack of research on QoL in patients with locally advanced gastric cancer. Therefore, future studies should focus on examining QoL outcomes in this patient group, especially in the context of comparing PG and TG in LAPGC.

Nevertheless, several limitations of this study should be acknowledged. First, although PSM improves group comparability, it cannot eliminate all biases inherent in retrospective studies. Thus, our findings should be interpreted as hypothesis-generating rather than definitive evidence. Second, we used the GRADE framework to assess key outcomes such as OS and RFS, which were rated as low-certainty due to study design and imprecision. Third, very few published reports provided outcome data stratified by minimally invasive surgical approach (laparoscopic vs. robotic) or by receipt of neoadjuvant therapy. Hence, the prespecified subgroup analyses could not be performed, leaving the influence of these factors on perioperative and oncologic end points uncertain. These findings underscore the importance of more detailed and standardized data reporting in future research, particularly in multi-institutional studies and real-world datasets.

## Conclusion

In conclusion, PG appears to be a feasible alternative to TG, potentially providing better short-term outcomes without compromising long-term survival in patients with LAPGC. However, large-scale, multicenter, prospective randomized controlled trials are needed to confirm these findings and provide more definitive guidance for clinical decision-making.

## Data Availability

The original contributions presented in the study are included in the article/**Supplementary Material**. Further inquiries can be directed to the corresponding author.
