# Supplementary figures and images for: Proximal vs. total gastrectomy for proximal advanced gastric cancer: a systematic review and meta-analysis of propensity score-matched studies
Source: Front Oncol. 2025 Sep 26;15:1632011. doi: 10.3389/fonc.2025.1632011 (PMC12510815; doi:10.3389/fonc.2025.1632011)

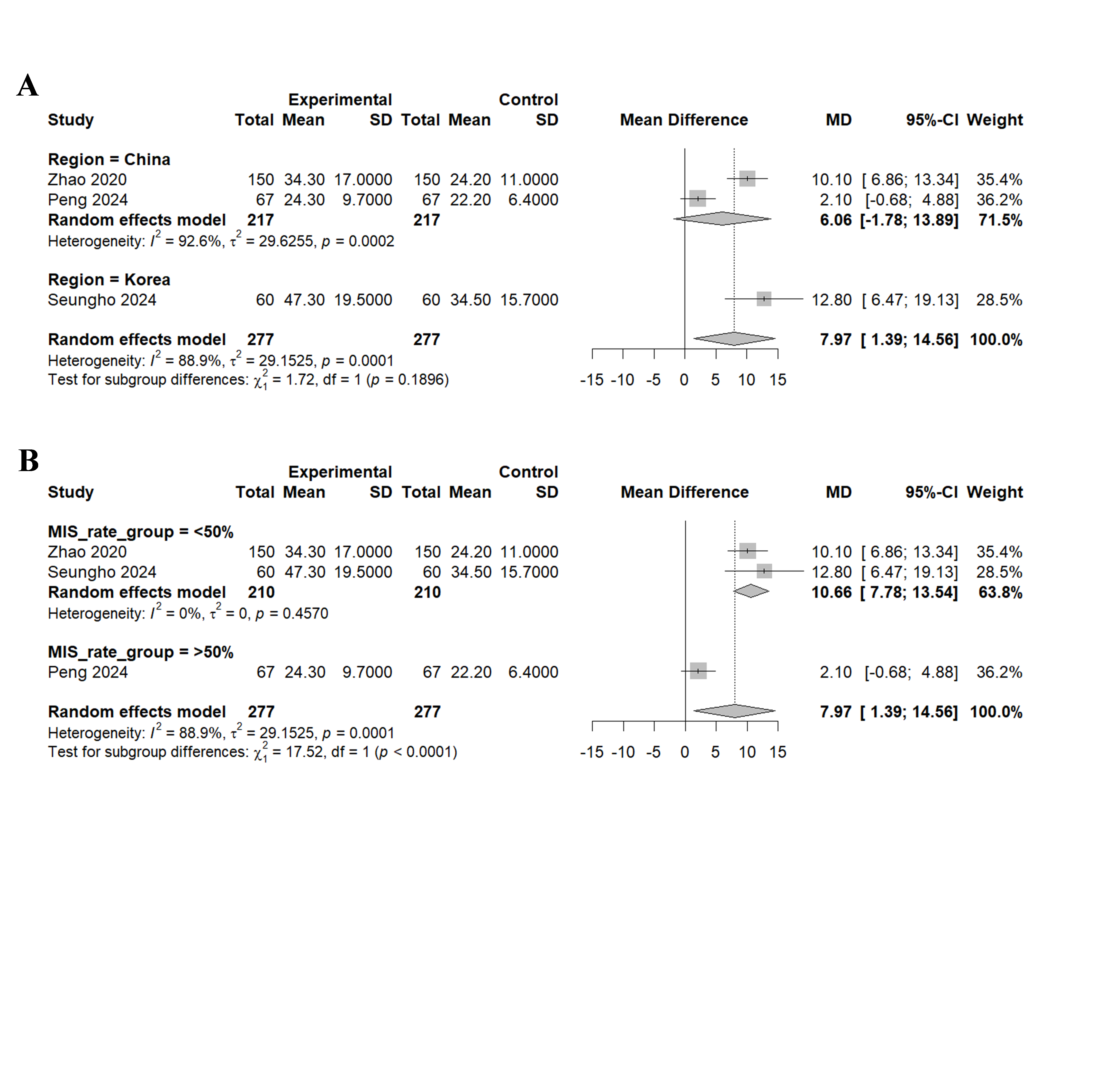

Supplement: Supplementary file 1 [file Image1.tif]
