# Supplementary material for: Proximal vs. total gastrectomy for proximal advanced gastric cancer: a systematic review and meta-analysis of propensity score-matched studies
Source: Front Oncol. 2025 Sep 26;15:1632011. doi: 10.3389/fonc.2025.1632011 (PMC12510815; doi:10.3389/fonc.2025.1632011)
Supplement: Supplementary file 2 [file Table1.docx]

| **Full Search Strategy** |
| --- |
| **Database: PubMed**  **Search Date**: Up to December 31, 2024  **Search String**: ("proximal gastric cancer"[Title/Abstract] OR "upper third gastric cancer"[Title/Abstract] OR "adenocarcinoma of esophagogastric junction"[Title/Abstract] OR "AEG"[Title/Abstract] OR "proximal advanced gastric cancer"[Title/Abstract]) AND ("proximal gastrectomy"[Title/Abstract]) |
| **Database: EMBASE**  **Search Date**: Up to December 31, 2024  **Search String**: ('proximal gastric cancer':ab,ti OR 'upper third gastric cancer':ab,ti OR 'adenocarcinoma of esophagogastric junction':ab,ti OR 'AEG':ab,ti OR 'proximal advanced gastric cancer':ab,ti) AND ('proximal gastrectomy':ab,ti) |
| **Database: Cochrane Library**  **Search Date**: Up to December 31, 2024  **Search String**: ("proximal gastric cancer" OR "upper third gastric cancer" OR "adenocarcinoma of esophagogastric junction" OR "AEG" OR "proximal advanced gastric cancer") AND ("proximal gastrectomy"): in Title, Abstract, or Keywords |
