# Supplementary material for: Proximal vs. total gastrectomy for proximal advanced gastric cancer: a systematic review and meta-analysis of propensity score-matched studies
Source: Front Oncol. 2025 Sep 26;15:1632011. doi: 10.3389/fonc.2025.1632011 (PMC12510815; doi:10.3389/fonc.2025.1632011)
Supplement: Supplementary file 3 [file Table2.docx]

**Supplementary Table 2.** GRADE Evidence Profile for OS and RFS.

| **Outcome** | **No. of Studies** | **Study Design** | **Risk of Bias** | **Inconsistency** | **Indirectness** | **Imprecision** | **Publication Bias** | **Overall Certainty of Evidence** | **Comments** |
| --- | --- | --- | --- | --- | --- | --- | --- | --- | --- |
| OS | 4 | PSM | Serious ^a^ | Not serious | Not serious | Serious ^b^ | Undetected | Low | All studies were retrospective and single-center; wide confidence intervals in pooled effect. |
| RFS | 2 | PSM | Serious ^a^ | Not serious | Not serious | Serious ^b^ | Undetected | Low | Limited number of studies; imprecise estimates due to small sample size. |

Abbreviations: OS, Overall Survival; PSM, Propensity Score Matching; RFS, Recurrence-Free Survival

^a^ All included studies were retrospective and observational in nature, despite using PSM to reduce bias.

^b^ Total sample size was small; confidence intervals were wide and some estimates crossed the line of no effect.
